# Unusual cutaneous Langerhans cell sarcoma without extracutaneous involvement

**DOI:** 10.1186/1746-1596-8-20

**Published:** 2013-02-06

**Authors:** Yang Li, Bin Li, Xiao-ying Tian, Zhi Li

**Affiliations:** 1 Department of Pathology, The First Affiliated Hospital, Sun Yat-sen University, 58, Zhongshan Road II, Guangzhou, 510080, China; 2 School of Chinese Medicine, Hong Kong Baptist University, 7, Baptist University Road, Kowloon Tong, Hong Kong, China

**Keywords:** Langerhans cell sarcoma (LCS), Langerhans cell tumor, Cutaneous involvement, Differential diagnosis

## Abstract

**Abstract:**

Langerhans cell sarcoma (LCS) typically presents as cytologic atypia and clinical aggressiveness and may involve multiple organs during the progression of the disease. Primary skin LCS without any extra-cutaneous site association is extremely rare and only a few such cases have been described in the literature. We present a case of unusual primary LCS in skin occurring in a middle-aged male patient. Physical examination revealed a large ulcerated cutaneous lesion and a smaller nodular lesion were located in the skin of the extensor side of his right knee. There was no regional lymph node or any other extra-cutaneous organ involvement. Histologically, typical large and pleomorphological tumor cells with epithelioid appearance and significantly malignant cytological features were observed to infiltrate in dermis and subcutaneous tissue. By immunohistochemistry, the tumor cells were positive for CD1a, S-100 protein and largerin strongly and diffusely. However, these cells were negative for CD3, CD20, CD21, pan-cytokeratin, HMB-45, Melan-A, and MPO. A diagnosis of primary cutaneous LCS was made. The patient received systemic chemotherapy of CHOP regimen, and was on a regular follow-up period for 12 months. There was no sign of relapse of tumor or any other extra-cutaneous organ involvement by whole body positron emission tomography/computed tomography (PET/CT) study. Because LCS is a high-grade malignancy with poor prognosis, it suggests that strict histological analysis and thorough radiographic examination are necessary for accurately diagnosing this tumor even if cutaneous involvement presented only.

**Virtual slides:**

http://www.diagnosticpathology.diagnomx.eu/vs/6527428618381393

## Background

According to the degree of cytologic atypia and clinical aggressiveness, Langerhans cell tumors are divided into two main subgroups: Langerhans cell histiocytosis (LCH) and Langerhans cell sarcoma (LCS) [1]. LCS is a rare high-grade neoplasm with overtly malignant cytologic features and aggressive clinical course. It can occur at any age and may involve multiple systems or tissues. Skin and underlying soft tissue are the most common involvement, but other unusual locations, such as bone [2], lung [3], gallbladder and peritoneal lymph nodes [4] have also been described in the literature. LCS may be limited to cutaneous involvement or may progress to affect other organs. However, to our knowledge, primary cutaneous LCS without any extra-cutaneous association is extremely rare and so far only a few such cases with immunohistochemical and/or ultrastructural confirmation have been described in the literature [5,6]. Due to its rareness in skin, the accurate diagnosis of cutaneous LCS could be difficult to be made because similar microscopic features may be encountered in metastatic cancer, malignant melanoma, anaplastic large cell lymphoma and myeloid sarcoma. Therefore, it is indeed a challenge for clinicians to make a correct diagnosis when the LCS presents as a solitary cutaneous mass because inaccurate diagnosis may lead to improper treatment. Herein we describe an additional case of primary cutaneous LCS arising in the skin in a middle-aged male patient without any extra-cutaneous manifestation in order to provide valuable information in this field.

## Case presentation

### Clinical presentation and management

**Figure 1:**
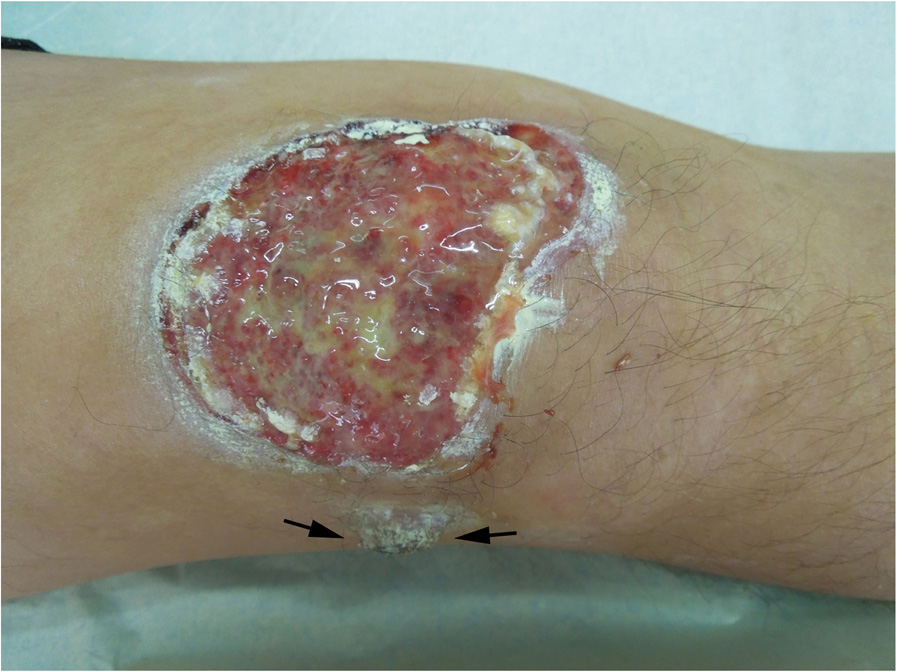
**Physical examination showed a 8.0 cm×6.0 cm well-defined ulcerated lesion in the skin of the extensor side of his right knee.** A solitary nodular lesion, measuring 1.5 cm in diameter was also found near the large ulcerated lesion (black arrow).

A 48-year-old Chinese male visited his local hospital in May 2010 with a gradually enlarged and ulcerated erythematous plaque on his right knee. The patient stated that the lesion had been presented on the extensor side of his right knee for approximately 6 months and treated with a steroid ointment initially. However, the lesion gradually enlarged and ulcerated, and a new skin nodule presented with pain during the last few months. As a result, the patient was referred to our hospital for examination and treatment. Physical examination showed 8.0 cm×6.0 cm well-defined ulcerated plaque on the extensor side of his right knee. A solitary nodular mass, measuring 1.5 cm in diameter was also found near the large ulcerated lesion (Figure 1). There was no weight loss and no palpable lymphadenopathy or organomegaly. The laboratory results, including blood count, differential, liver and renal function, were within the normal range. A clinically presumed diagnosis was squamous cell carcinoma of skin. Surgical resection of the skin lesion without preoperative skin biopsy was performed. After diagnosis, the patient underwent polychemotherapy according to a CHOP protocol (cyclophosphamide, doxorubicin, oncovin, and prednisone) for six cycles. The skin ulcerated lesion regressed and the nodule became smaller in size. The patient was on a regular follow-up period for 12 months after chemotherapy. The bone marrow examination was performed 6 months after chemotherapy, but there was no abnormality found. Since there was a possibility of multiple organs involvement, the patient was referred to a whole body positron emission tomography/computed tomography (PET/CT) study to search the potential secondary tumor, but no abnormality was found. During the period of follow-up, there was no sign of recurrence of tumor and lymph node enlargement.

## Material and methods

The surgical specimens, including large ulcerated plaque and small nodular mass, were routinely fixed in 10% neutral buffered formalin. The tissues were embedded in paraffin. Four micrometer-thick sections were stained with H&E. Immunohistochemical analyses were performed using the ChemMate Envision/HRP Kit (Dako, Glostrup, Denmark). The antibodies used in this study included a broad panel of antibodies against CD1a, CD3, CD5, CD20, CD21, CD30, CD38, CD56, CD68, CD79a, ALK, S-100 protein, Langerin, pan-cytokeratin, HMB-45, Melan-A, MPO and ki-67. The antibodies were obtained from Dako Cytomation (Dako, Glostrup, Denmark), Santa Cruz Biotechnology (Santa Cruz, CA, USA) and Novocastra laboratories LTD (Hong Kong, China).

For detection of Epstein-Barr virus (EBV) infection in the tissues, in situ hybridization for EBERs (EBV-encoded RNAs) was performed. The EBERs detection kit was purchased from Dako (Glostrup, Denmark). The detection process was conducted according to the manufacturer’s instructions.

## Pathological findings

**Figure 2:**
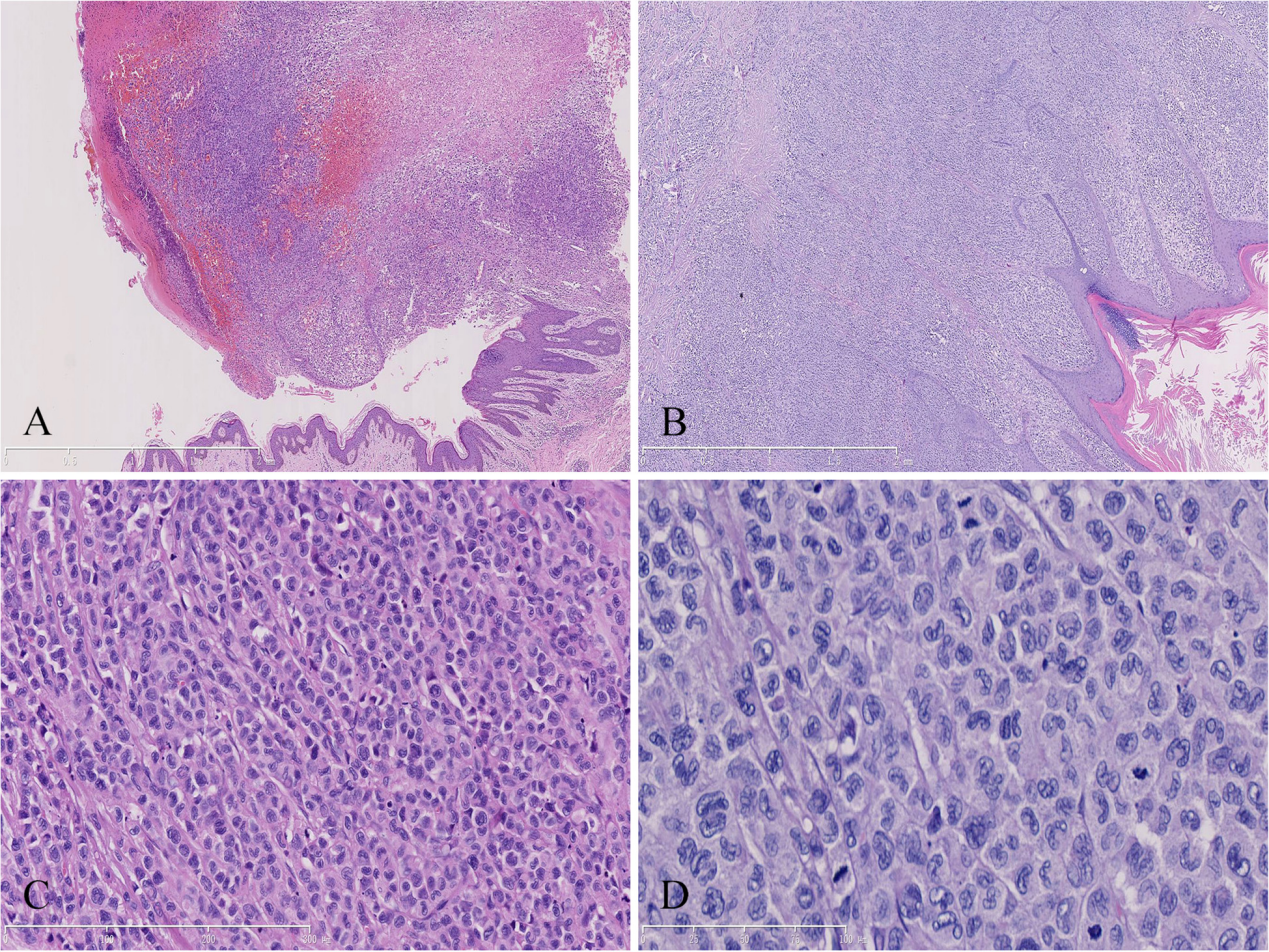
**Photomicrographs of the cutaneous lesions.** (**A**) Under low-power microscopic examination, the ulcerated lesion showed erosion of epidermis and diffuse infiltrating of large tumor cells in dermis and subcutaneous tissue. (**B**) The nodular cutaneous lesion was characterized by sheets of tumor cells in the dermis but the epidermotropism was not appreciated in the lesion. (**C**) Tumor cells were large epithelioid with large oval to convoluted nuclei and nuclear groves. (**D**) At high-power fields, the tumor cells exhibited significantly malignant cytological features with ill-defined cell borders and active mitotic figure (**A** and **B**, HE staining with original magnification ×40; **C**, HE staining with original magnification ×200; **D**, HE staining with original magnification ×400).

**Figure 3:**
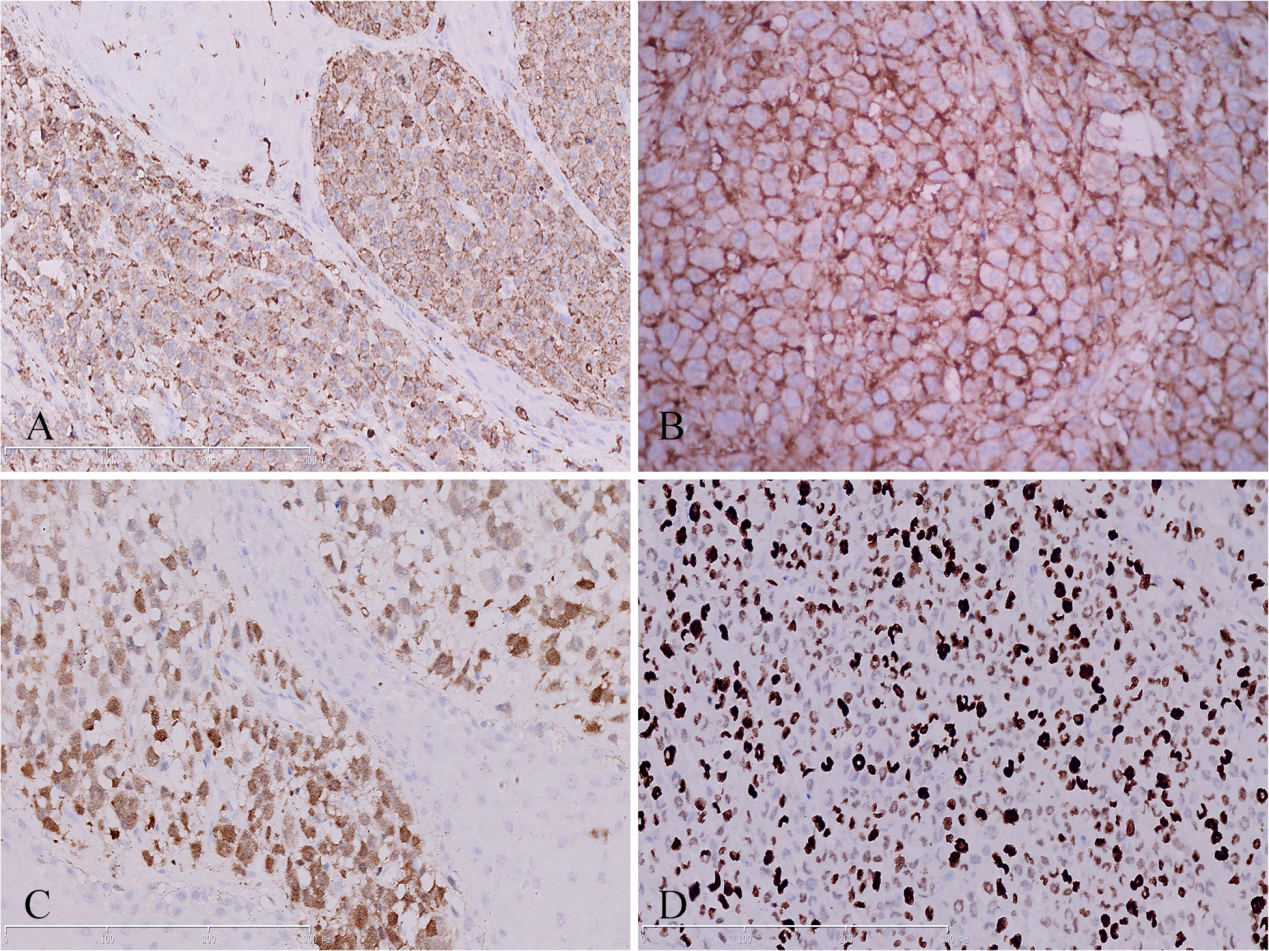
**Immunohistochemical analysis of the cutaneous lesions showed tumor cells were diffusely positive for largerin (A), CD1a (B) and S-100 protein (C).** (**D**) Ki-67 proliferating index of tumor cells was high approximately 80%positive (**A**-**D**, immunohistochemical staining with original magnification ×400).

Under microscopic examination, the ulcerated lesion and solitary skin nodule shared similar histopathological appearance characterized by diffusely infiltrating of large tumor cells in dermis and subcutaneous tissue but epidermotropism was not appreciated. The tumor cells exhibited large epithelioid appearance with abundant eosinophilic cytoplasm and ill-defined cell borders. They had large oval to convoluted nuclei with nuclear groves and dispersed chromatin. These cells showed significantly malignant cytological features with active mitotic figure and multinuclear giant cells. Infiltrated neutrophils, eosinophils and lymphocytes could also been observed (Figure 2). The immunohistochemistry staining showed that the tumor cells were diffusely positive for S-100, CD1a, langerin and CD68, but negative for CD3, CD5, CD20, CD21, CD30, CD38, CD56, CD79a, ALK, HMB-45, Melan-A, pan-cytokeratin and MPO. Ki-67 proliferating index was high approximately 80% (Figure 3). EBERs detection demonstrated that there was no EBV infection in tumor cells. Taken together, these morphological and immunological data were consistent with a cutaneous LCS according to the WHO diagnostic criteria [1].

## Conclusions

Langerhans cell sarcoma (LCS) is a rare but distinct tumor derived from dendritic cell lineage. It was firstly recognized by Wood and colleagues in 1984 as malignant histiocytosis X to describe a rapidly fatal cutaneous mass with massive tumor infiltration of multiple organs occurring in an elderly male patient [7]. In 1992, Tani *et al.* defined it as a “malignant neoplasm of Langerhans cells with the following criteria: (i) proliferation of typical Birbeck granule-containing tumor cells, and (ii) malignant cytological features such as atypia and frequent mitotic figures” [8]. The classification of tumor of dendritic cells is indeed controversial, and there are overlapped features among various entities of the histiocytic and dendritic cell lesions, making definitive diagnosis difficult [1]. According to the WHO classification, tumors of dendritic cell lineage include follicular dendritic cell tumors, interdigitating dendritic cell tumors, Langerhans cell tumors and other rare dendritic cell tumors. Langerhans cells and interdigitating dendritic cells share a common hematopoietic CD34+ precursor; in contrast, follicular dendritic cells do not have a hematopoietic origin. Langerhans cell tumors show expression of both CD1a and S-100 protein, however, interdigitating cells are positive for S-100 but negative for CD1a. Follicular dendritic cells express CD21 consistently, but never express CD1a. These specific immunophenotypes of tumor cells may be helpful to identify the accurate diagnosis of dendritic cell tumors. However, indeterminate dendritic cell tumor, a rare type of dendritic cell tumor and originate from the alleged precursor cells of Langerhan cells, is indeed difficult to distinguish from Langerhans cell tumor because both tumors consistently express S-100 protein and CD1a. By definition, indeterminate dendritic cells lack Birbeck granules on ultrastructural examination [9]. However, not all cases diagnosed as Langerhans cell tumors had ultrastructural studies to confirm the presence of Birbeck granules. Ben-Ezra *et al.* reported that only 3 cases presented Birbeck granules among 9 cases of LCS [10]. Deng *et al.* also described a cutaneous LCS without Birbeck granules and doubted it might be an indeterminate cell sarcoma [11]. Therefore, it is possible that some previously reported LCS might be confused with indeterminate cell neoplasms, and indeterminate cell neoplasms are actually underreported.

Recent studies suggest that langerin (CD207) represents a very specific marker for Langerhans cells and derived tumors [4,12], and it has been negative in reported indeterminate cell neoplasms [13,14]. Langerin is a type II Ca^2+^-dependent lectin and induces the formation of Birbeck granules, the presence of which supports a Langerhans cell origin. However, Wang *et al.* recently demonstrating a langerin-positive LCS did not indicate Birbeck granules in the cytoplasm of the neoplastic cells on ultrastructural examination [15]. Verdijk *et al.* found a lack of Birbeck granules in Langerhans cells to be associated with a mutation in the langerin gene [16]. The lack of Birbeck granules under the electron microscopy might be due to the damage of Birbeck granules during histological process [3] or poor differentiation of tumor cells [10]. Thus, langerin is a useful marker for distinguishing Langerhans cell tumors from indeterminate cell tumors even in Birbeck granules-negative cases [12,17]. In the present case, we found that the tumor cells exhibited remarkably cellular atypia and higher mitotic activity with co-expression of CD1a, S-100 protein and langerin. This is consistent with a typical primary LCS in spite of the presence of Birbeck granules in tumor cells.

Despite its enigmatic histogenesis, LCS is an exceedingly rare tumor. To the best of our knowledge, only 30 LCS cases (including the present case) have been reported in the English-language literature to date [2-8,11,15,18-24]. The age at the time of diagnosis ranged from newborn [10] to 81 years old [3] without gender bias. LCSs show a multiple organs involvement. Most of the patients had lymph node and skin involvement, although the lung, bone, mediastina, liver, spleen and heart can also be involved. The skin was the only organ involved in a few cases [5,6]. Although most LCSs exhibited classical morphological and immunohistochemical features, individual cases showed unusual appearances, such as aberrant cytoplasmic CD3 expression and involving epidermis [15,25]. LCSs are high-grade malignancy and show aggressive clinical course. Chemotherapy regimens have been demonstrated to be helpful to slow progression of LCS in various degrees. Radiotherapy has also been applied in several cases, but the effects remain uncertain. From the patients for whom follow-up data are available, 53.3% (16/30) died of their disease within 2 years despite conventional combination chemotherapy, surgery, and radiotherapy. However, 2 patients of 3 reported LCSs with only cutaneous involvement survived and were alive in complete remission (Table 1). In the present case, we found that the patient achieved a partial remission after chemotherapy and extra-cutaneous manifestation was not observed during the period of follow-up, although the follow-up period was not very long for the patient after therapy. These findings indicate that primary cutaneous LCSs without multiple organs involvement might have a somewhat smoldering stage. Once the tumor progress, dissemination of extracutaneous sites might be presented and the patient might gain a poor prognosis with aggressive clinical course. A recent study has demonstrated that some cases of histiocytic sarcomas do not pursue an aggressive clinical course, particularly cases with clinically localized disease, and the tumor size appears to be an important prognostic factor [26]. In addition, Kawase *et al.* suggested that CD56+ Langerhans cell neoplasms share an aggressive clinical behavior and a worse outcome [2]. For our reported patient, the tumor was localized but was large in size. There was no CD56 expression in tumor cells. It should carry an unfavorable prognosis. Of course, long-term follow-up should be performed to verify this postulation.

**Table 1:** Clinicopathological features of Langerhans cell sarcoma with only cutaneous involvement described in present and previous reports

**No.**	**Authors (yr.)**	**Diagnosis**	**Age (year)/Gender**	**Clinical presentation**	**Immunophenotype**	**EM/MA**	**Treatment**	**Outcome**
1	Pileri SA (2002) [5]	Langerhans cell sarcoma	50/Female	Multiple nodular lesions on skin	CD1a+/S-100+/CD68+/lysozyme+/CD20-/CD21-/CD45-/CD3-/CD30-/MPO-/EMA-	EBERs-	NA	NA
2	Pileri SA (2002) [5]	Langerhans cell sarcoma	10/Female	Single nodular lesion on skin	CD1a+/S-100+/CD68+/CD20-/CD21-/CD45-/CD3-/CD30-/CD34-/MPO-/EMA-	EBERs-	Surgery and radiotherapy	Alive in complete remission
3	Misery L (2003) [6]	Malignant Langerhans cell tumor	38/Female	Single red, hardened nodular lesion on skin	CD1a+/S-100+/CD68+/HAM56+/lysozyme+/CD45-/HMB45-/EMA-/CK-/CD20-/CD79-	Birbeck granules+	Surgery with large excision	2-year follow-up, no relapse/alive
4	The present case	Langerhans cell sarcoma	48/male	Ulcerated and nodular lesion on skin	CD1a+/S-100+/langerin+/CD68(focal)+/CD56-/CD3-/CD20-/CD21-/MPO-/CK-	EBERs-	Surgery and chemotherapy with CHOP regimen	PR, 1-year follow-up, no relapse/alive

*EM*, electron microscopy; *MA*, molecular analysis; *EMA*, epithelial membrane antigen; *CK*, cytokeratin; *MPO*, myeloperoxidase; *EBERs*, Epstein-Barr virus-encoded small RNAs; *PR*, partial remission; *NA*, not available.

As its rareness in skin and poorly differentiated morphologic features, cutaneous LCS should be differentially diagnosed from other epithelial or mesenchymal malignancy of skin, including metastatic cancer, malignant melanoma, anaplastic large cell lymphoma and myeloid sarcoma. All of these diseases exhibit skin lesion and a frankly malignant cytologic appearance with highly aggressive clinical course and a poor prognosis, which may sometimes cause diagnostic confusion with LCS. However, cutaneous squamous cell carcinoma or metastatic cancer shows an obvious nest structure with epithelial phenotype, such as pan-cytokeratin, CK7 or CK20. Melanoma might share S-100 protein positivity, but also expresse other melanocytic markers, such as HMB45 and melan-A. Anaplastic large cell lymphomas are CD30+ and EMA+ and may show ALK positivity. Occasionally, myeloid leukemia can firstly present in skin, in which CD68 and lysozyme positivity could be observed and sometimes difficult to be distinguished from LCS. The presence of myeloid specific markers, such as MPO, CD117, CD99 and CD34 should be helpful in making a diagnosis of myeloid leukemia first presenting in skin or cutaneous myeloid sarcoma. In our case, the tumor cells strongly expressed CD1a, S-100 protein and langerin, which were Langerhans cell-specific marker, arguing against other cutaneous hematological neoplasma.

In conclusion, LCSs are extremely rare and only a few cases of LCSs with only cutaneous involvement have been reported in the literature. Our additive case also presented its rarity of site. The diagnosis of primary cutaneous LCS is difficult and should be made cautiously, particularly for those cases with clinically localized disease, but lacking the extra-cutaneous manifestation. LCS is a highly aggressive hematopoietic malignancy with poor prognosis. We suggest a long period of follow-up is necessary and radiographic examination is helpful for demonstrating organ involvement to inspect the progression even if the patient had complete remission at initial chemotherapy.

## Consent

Written informed consent was obtained from the patient for publication of this case report and any accompanying images. A copy of the written consent is available for review by the Editor-in-Chief of this journal.

## Abbreviations

LCH: Langerhans Cell Histiocytosis; LCS: Langerhans Cell Sarcoma; EBV: Epstein-Barr virus; EBERs: EBV-Encoded RNAs.

## Competing interests

The authors declare that they have no competing interests.

## Authors’ contributions

YL and BL made contributions to acquisition of clinical data, and analysis of the histological and immunohistochemical features. They are co-first authors, and have an equal contribution to this work. XYT drafted the manuscript. ZL revised manuscript critically for important intellectual content and had given final approval of the version to be published. All authors read and approved the final manuscript.
